# Insights into the posttranslational structural heterogeneity of thyroglobulin and its role in the development, diagnosis, and management of benign and malignant thyroid diseases

**DOI:** 10.1590/2359-3997000000103

**Published:** 2016-01-01

**Authors:** Ana Carolina W Xavier, Rui M. B. Maciel, José Gilberto H Vieira, Magnus R. Dias-da-Silva, João R. M. Martins

**Affiliations:** 1 Departamento de Medicina Escola Paulista de Medicina Universidade Federal de São Paulo São Paulo SP Brasil Laboratório de Endocrinologia Molecular e Translacional, Divisão de Endocrinologia e Metabologia, Departamento de Medicina, Escola Paulista de Medicina, Universidade Federal de São Paulo (EPM/Unifesp), São Paulo, SP, Brasil; 2 Departamento de Medicina Faculdade de Medicina (Famed) Universidade Federal do Mato Grosso do Sul Campo Grande MS Brasil Clínica Integrada V, Endocrinologia e Metabologia, Departamento de Medicina, Faculdade de Medicina (Famed), Universidade Federal do Mato Grosso do Sul (UFMS), Campo Grande, MS, Brasil; 3 Departamento de Bioquímica EPM Unifesp São Paulo SP Brasil Divisão de Biologia Molecular, Departamento de Bioquímica, EPM/Unifesp, São Paulo, SP, Brasil

**Keywords:** Thyroglobulin, posttranslational protein modifications, heterogeneity, thyroid diseases

## Abstract

Thyroglobulin (Tg) is the major glycoprotein produced by the thyroid gland, where it serves as a template for thyroid hormone synthesis and as an intraglandular store of iodine. Measurement of Tg levels in serum is of great practical importance in the follow-up of differentiated thyroid carcinoma (DTC), a setting in which elevated levels after total thyroidectomy are indicative of residual or recurrent disease. The most recent methods for serum Tg measurement are monoclonal antibody-based and are highly sensitive. However, major challenges remain regarding the interpretation of the results obtained with these immunometric methods, particularly in patients with endogenous antithyroglobulin antibodies or in the presence of heterophile antibodies, which may produce falsely low or high Tg values, respectively. The increased prevalence of antithyroglobulin antibodies in patients with DTC, as compared with the general population, raises the very pertinent possibility that tumor Tg may be more immunogenic. This inference makes sense, as the tumor microenvironment (tumor cells plus normal host cells) is characterized by several changes that could induce posttranslational modification of many proteins, including Tg. Attempts to understand the structure of Tg have been made for several decades, but findings have generally been incomplete due to technical hindrances to analysis of such a large protein (660 kDa). This review article will explore the complex structure of Tg and the potential role of its marked heterogeneity in our understanding of normal thyroid biology and neoplastic processes.

## INTRODUCTION

Thyroglobulin (Tg) is a high molecular mass protein produced exclusively by the thyroid follicular cells. Measurement of Tg levels in serum is of great clinical importance in medical practice, as part of the diagnostic workup for several benign conditions, but especially in the postoperative follow-up of differentiated thyroid cancer (DTC).

Under normal conditions, only a very small amount of Tg bypasses intrathyroidal proteolysis and reaches the bloodstream in essentially intact form. Therefore, only with the advent of radioimmunoassay techniques in the late 1960s did measurement of the minute concentrations of Tg occurring in human serum become possible (1,2). This method was brought into routine use in the early 1970s, with the studies of Van Herle and cols. (3). However, the advent of monoclonal antibodies led to a great progress in this field, enabling the development of more modern, automated, non-isotopic, two-site assays. These techniques revolutionized DTC follow-up, because of their high sensitivity (4) and their ability to detect concentrations of Tg as low as 0.1 ng/mL. This fact has made serum Tg measurement a cornerstone of post-thyroidectomy follow-up of patients with DTC, as Tg levels contribute to the definition of cure, residual disease, and even tumor recurrence (5,6).

Although modern assays based on monoclonal anti-Tg antibodies (TgAb) have enabled the development of highly sensitive and specific measurement techniques, some pitfalls persist in clinical practice. One of these drawbacks would be the variability between assays using different monoclonal antibodies, which may cause Tg levels to have an up to two-fold difference when the same sample is analyzed by different assays, even in the absence of endogenous TgAb (4). In addition, some assays may recognize differences between normal Tg and tumor-derived Tg (7). With the majority of current assays, the presence of endogenous TgAb in the serum of patients with DTC can interfere with test results, usually producing low and/or undetectable Tg levels, which limits the use of serum Tg as a marker for follow-up of these patients (8,9). The exact mechanism of such interference is still unknown but may involve steric hindrance and/or masking of epitopes, events that hinders the recognition of Tg by reporter antibodies (10). This limitation is a particular concern because 25-30% of patients with DTC have circulating TgAb, a three times greater prevalence than that found in the general population (4). This gives rise to some pertinent speculation: a) could tumor Tg be structurally anomalous and, consequently, more immunogenic? Or b) could the “economy” of tumor cells, which are more prone to proliferation and expansion, drive production of “naked” Tg, with simplified or even absent posttranslational modifications (iodination, glycosylation, sulfation) and, thus, bearing a greater number of exposed antigenic sites?

Attempts to elucidate the structure of Tg date back to the 1950s. Since then, evidence has arisen that serum Tg is composed of a pool of polypeptide fragments which are highly heterogeneous in terms of size, but also exhibit differences in iodine content and in the distribution of glycosylation, and sulfation (11,12). However, technical limitations at that time, compounded by the very high molecular mass of Tg, made these initial studies somewhat incomplete. A critical step toward a better understanding of the structure of Tg was made in 1985, when its peptide sequence was described (11). This enabled new discoveries, such as the identification of new posttranslational modifications to its primary structure, and contributed to the development and enhancement of immunoassays for its detection. More recently, the “omics” era has created new perspectives for expansion of our knowledge of the structure of Tg, but many challenges remain (13). This review article will explore several aspects of Tg biosynthesis, with an emphasis on posttranslational processes, and their potential role in the development, diagnosis, and management of thyroid diseases.

## STRUCTURE OF THYROGLOBULIN

Tg is synthesized in the endoplasmic reticulum of the follicular cells, modified in the Golgi complex and secreted into the follicular lumen. The gene that codes for Tg has been mapped to chromosome 8. It is fairly conserved across mammal species, with some sequence homology between human Tg and that of other animals (11). Tg is known to being enhanced with species development. Recent comparative phylogenetic analyses of Tg from echinoderms, amphibians, zebrafish, and vertebrates provide evidence of the continued evolution of this protein (14).

Tg messenger RNA codes for a polypeptide chain consisting of 2,768 amino acid residues that will compose the backbone of a high molecular weight glycoprotein made up of two 330-kDa polypeptide chains. Mature Tg (660 kDa) is formed by linking two disulfide-bonds polypeptide chains (15,16).

**Figure 1:**
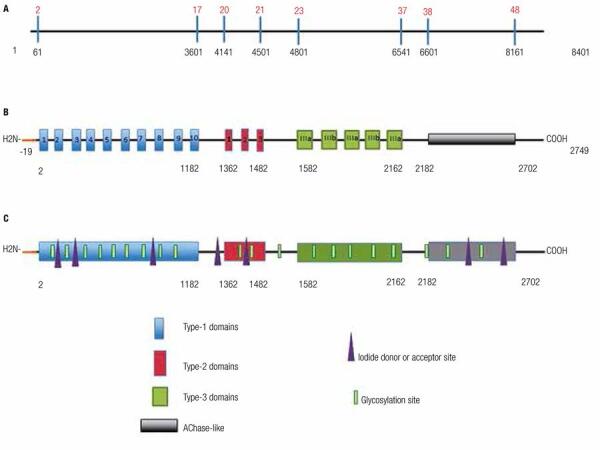
Schematic representation of the TG gene and polypeptide chain of human thyroglobulin (Tg). (A) Structure of the TG gene. Nucleotides are shown in black, and exons coding for the repeat domains and C-terminal portion are shown in red. (B) Structure of the Tg polypeptide chain (2749 amino acids). The signal peptide is shown in orange at the N-terminal portion, which is well-organized and divided into 3 major domains: type 1, encoded by exons 2-17 and composed of 10 repeats of a 50-amino acid sequence; type 2, encoded by exons 20-21 and composed of three repeats of 14-17 amino acid residues; and type 3, encoded by exons 23-37 and composed of 2 subtypes, a and b, which repeat 5 times and contain approximately 100 amino acids each. The C-terminal portion is not similarly organized and exhibits sequence homology to proteins in the acetylcholinesterase family (AChase-Like). (C) Schematic representation of the polypeptide sequence. Glycosylation sites are shown in green and iodination sites represented by purple triangles.

Tg is divided into two regions: N-terminal and C-terminal (Figure 1). These regions are so distinct that it has been suggested that the Tg gene was formed by the fusion of two different ancestral genes (17).

The first 19 residues of the N-terminal end constitute the signal peptide, which directs the pre-polypeptide during posttranslational processing and secretion into the Golgi complex (9,14) (Figure 1).

Analysis of the polypeptide chain at the N-terminal end (1-2170) reveals an organized structure (Figure 1).

There are three domain types in which the positions of cysteine residues are highly conserved. By forming disulfide bonds between monomers, these residues increase molecular stability and resistance to proteolysis (14). These domain types are: type 1, composed of approximately 50 residues which repeat 10 times between amino acids 1 and 1200; type 2, composed of 14-17 residues that repeat three times between positions 1436 and 1483; and type 3, which is composed of two subtypes (A and B) that repeat five times between positions 1583 and 2170 (16). It has been speculated that each of these regions has a different function, with region 1 controlling Tg binding to other proteins; region 2 controlling cell adhesion; and region 3 playing a structural role (14).

Conversely, the C-terminal region (approximately 581 residues) does not exhibit internal homology, but it is similar to a protein in the acetylcholinesterase family (carboxylesterase, type B) (17). Furthermore, this part of the molecule has reduced cysteine and increased tyrosine content as compared with the rest of the monomer. Studies have shown that the acetylcholinesterase-like region works as a dimerization domain to facilitate intracellular transport (18) (Figure 1).

## TG AND THYROID HORMONOGENESIS

Tg accounts for over 75% of all thyroid proteins, and it is present in the colloid and in much of the intracellular material of the gland (16).

The physiologic functions of Tg include providing a matrix for thyroid hormone synthesis and storing iodine in the form of the iodinated tyrosyl residues monoiodotyrosine (MIT) and diiodotyrosine (DIT), which are the precursors of the thyroid hormones T3 (triiodothyronine) and T4 (thyroxine). In addition, Tg also plays a role in modulating the expression of genes involved in the synthesis of other thyroid proteins (sodium-iodide symporter [NIS] and thyroid peroxidase [TPO]) and transcription factors involved in normal thyroid physiology (TTF-1, TTF-2, and PAX-8) (19,20).

In addition to these physiologic roles, Tg is involved in the pathogenesis of several thyroid diseases, particularly autoimmune thyroiditis (in which it acts as an antigen) and DTC; in patients with DTC, Tg is used as a marker of residual disease or tumor recurrence after total thyroidectomy (5,6).

**Figure 2:**
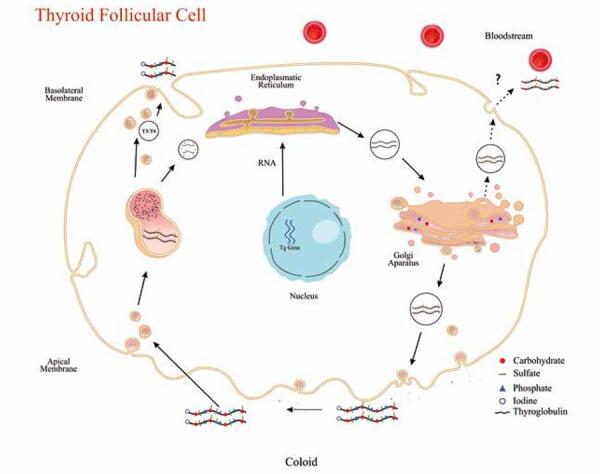
Schematic model of synthesis and posttranslational modifications of thyroglobulin (Tg) in the follicular cell. The two 330-kDa polypeptide chains, linked by disulfide bonds, are synthesized in the endoplasmic reticulum from mRNA transcribed from the TG gene, located on chromosome 8. Posttranslational modifications (glycation, sulfation, and phosphorylation) take place in the Golgi apparatus. Tg is then secreted into the colloid, where iodination occurs to form the thyroid hormone precursors MIT and DIT. Iodinated Tg enters the follicular cell cytoplasm by pinocytosis and combines with lysosomal vesicles containing proteolytic enzymes, which lyse Tg and release the thyroid hormones into the bloodstream. Part of the remaining material is reused by the cell.

As noted above, Tg is the matrix for thyroid hormone synthesis. This process begins with formation of the polypeptide chain within the endoplasmic reticulum and continues with posttranslational modifications, which occur simultaneously: glycosylation and sulfation (primarily in the Golgi complex), iodination (in the thyrocyte apical membrane), and phosphorylation (in a yet-unknown compartment, but possibly the Golgi complex) (21,22). The critical stage of thyroid hormone synthesis is iodination, in which Tg must be directed to the follicular lumen and bind to the apical membrane of the follicle, where tyrosyl residues will be iodinated in a reaction catalyzed by TPO activated by hydrogen peroxide. The lumen is also the site of the coupling reaction, whereby two DIT residues or one MIT and one DIT residues fuse to form T4 and T3 respectively. Iodinated Tg returns to the cell by endocytosis; Tg-containing intracellular vesicles fuse with lysosomes, where they will be acted upon by cathepsins, aminopeptidases, and dipeptidyl peptidases to release T4 and T3, which are then secreted into the bloodstream (23). This entire hormonogenesis process is regulated primarily by the action of thyroid-stimulating hormone (TSH) (Figure 2).

## POSTTRANSLATIONAL MODIFICATIONS TO TG

Tg undergoes extensive posttranslational modifications, including glycosylation, iodination, and phosphorylation. These modifications influence several aspects of normal Tg and thyroid hormone biosynthesis and play roles in various pathologic processes.

## GLYCOSYLATION

Approximately 10% of the molecular mass of Tg is composed of carbohydrate residues (21). The glycosylation process may occur via two basic mechanisms catalyzed by glycosyltransferases, namely, addition of N-linked and O-linked sugars. The bonding of sugars to Tg occurs step by step during transport of the nascent polypeptide chain from the endoplasmic reticulum to the Golgi complex (24) (Figure 2).

The major basic types of N-linked sugars are type A, or simple, which contain mannose and N-acetylglucosamine residues; and type B, or complex, which contain galactose, fucose, and sialic acid residues in addition to those of type A sugars. Other types include type C, which contain O-linked sugars, the residues of which are still largely unknown; and type D, which contain chains of the proteoglycan chondroitin sulfate. Type A and B sugars bind to Tg via asparagine residues, whereas type C and D sugars bind to serine residues (21).

Oligosaccharides play roles in several thyroid functions. Notable roles include: influence on iodination and hormone synthesis; specific interactions with microsomes and membrane receptors; directing Tg to subcellular and extracellular compartments (24); Tg immunoreactivity (25); and protein folding and recycling (26). Sialic acid does not participate in hormonogenesis, but it is essential for Tg binding to its transmembrane transporter and influences its solubility and immunoreactivity (27).

Regarding the immunoreactivity of Tg, Fenouillet and cols. compared the Tg secreted by cells in culture to that extracted from thyroid tissues and found that the former exhibited fourfold less binding to polyclonal antibodies against native Tg. This difference was attributed to the greater amount of complex branched carbohydrates on secreted Tg as compared with thyroid-derived Tg. These carbohydrate chains appear to interfere with antigen-antibody recognition by masking regions of the polypeptide chain that constitute the main antigenic determinants (25). Another possibility is that carbohydrates may distort the structure of the glycoprotein and thus weaken antigen-antibody interactions. Salabè and cols. also assessed the influence of glycosylation on Tg immunoreactivity, finding that immunoreactivity increases after desialylation, which suggests that sialic acid masks antigens (27).

Despite advances, much remains to be explored regarding Tg glycosylation.

## SULFATION

Sulfation is a late posttranslational modification that takes place in the Golgi complex (Figure 2). Sulfate residues are present in carbohydrate units and in some amino acids, especially tyrosine (28). The sulfate residues of Tg are essentially present in complex carbohydrates, particularly in chondroitin sulfate chains, an event that only occurs in human Tg (29).

The role of these sulfated residues varies. They are involved in molecular conformation and protein interactions, take part in activation and deactivation of biological activities, and account for much of the negative charge of the Tg molecule (10,30). However, these roles have yet to be fully elucidated and little is known regarding their regulation, although investigators have reported that TSH downregulates Tg sulfation (31).

A role of sulfate in tumor dissemination and growth mechanisms has been speculated. Loss of sulfate has been documented in some tumor models, including pancreatic cancer (32). Furthermore, Emoto and cols. (33) found decreased sulfation in Tg derived from papillary thyroid cancer (PTC) cells as compared with normal Tg, and believed this might influence tumor growth.

## PHOSPHORYLATION

Phosphorylation of Tg probably takes place in the Golgi complex, but its mechanism is still largely unclear (Figure 2). Studies conducted in the 1980s (22) found that approximately 50% of phosphate present in Tg is covalently bound to carbohydrate chains, 30% to serine residues, and the remaining 20% to tyrosine. The role of phosphorylation of sugar residues is unclear, but, by analogy to other proteins, phosphorylation of mannose residues may be involved in directing Tg through the Golgi complex to the endosomes and, finally, to the lysosomes, where it is degraded for iodothyronine release and recycling of amino acids (34). The role of serine and tyrosine residue phosphorylation is even less clear. *In vitro* studies conducted on FRTL-5 cells and using dog thyroid-derived Tg have suggested a potential role of this posttranslational modification in the Tg iodination process and in TSH-independent protein synthesis (35).

## IODINATION

Tg contains a variable amount of iodine, which depends on the availability of this element in the body. The percentage of iodine in Tg by mass ranges from 0.1% to 1% across several species (21). Iodine transport to the thyroid is a two-stage process. The first involves the sodium/iodide-symporter (NIS), which is found in the basolateral membrane and actively transports iodine from the bloodstream into the follicular cells. In the second stage, which involves pendrin, a protein found at the apical membrane, iodine is transported from the cytoplasm to the follicle lumen, where it is stored for later use in the synthesis of thyroid hormones (21).

Iodination takes place in the follicular lumen, specifically at the thyrocyte apical membrane, to which iodine is transported and where it is bound to tyrosine residues to form MIT or DIT. This reaction is catalyzed by TPO and requires hydrogen peroxide, which is generated by NADPH-dependent oxidase (21). As noted above, this is followed by the coupling reaction, also catalyzed by TPO, whereby one MIT and one DIT molecule fuse to form T3 or two DIT molecules fuse to form T4.

The structure of Tg contains approximately 130-140 tyrosine residues, only 25-40 of which undergo iodination, and even fewer of which take part in the coupling reaction (21). Studies of Tg in different species have reported four main sites of hormonogenesis (A-D) and three secondary sites (G, N, and F), with elevated sequence similarity among species. Utilization of these sites varies depending on the origin of Tg and it is influenced by the availability of iodine (21).

Iodination might play a role in the immunogenicity of Tg, as does glycosylation. A study showed that the affinity of specific anti-native Tg monoclonal antibodies decreases with a reduction in the iodine content of the protein (36). However, another study suggests that the iodine content of the Tg molecule may alter its conformation, changing the structure of its polypeptides and its antigenic characteristics according to the type of monoclonal antibody used for its detection (37). Furthermore, iodine is necessary for the recognition of certain antigens, and thus contributes to the pathogenesis of autoimmune thyroiditis (36). There is a certain degree of uncertainty about the difference between iodine content in tumor Tg compared with normal Tg. Tumor Tg has a much less amount of iodine than that of normal Tg, whereas there is no difference in the iodine content of serum Tg from patients with benign and malignant tumors when compared to that from normal subjects (38).

## TG HETEROGENEITY AND THYROID DISEASE

Studies have shown differences in Tg across several benign thyroid diseases. Saboori and cols. showed that normal Tg and thyroid-derived Tg from patients with Graves’ disease exhibit distinct profiles when analyzed by ion exchange chromatography. Unlike normal Tg, the Tg of Graves’ disease has a greater proportion of protein fragments with low affinity for ion exchange columns, a difference the authors attributed to the lower iodine content of these polypeptides. Furthermore, these under-iodinated forms of Tg showed decreased reactivity to monoclonal TgAb on Western blot analysis (39).

Other studies have demonstrated that different peptide segments of Tg react in specific ways with sera from patients with autoimmune thyroiditis. This suggests that the polypeptide structure of certain Tg fragments has a conformational arrangement associated with increased antigenicity, and may thus contribute to the pathogenesis of autoimmune thyroid diseases (40).

Regarding thyroid neoplasms, attempts to isolate distinct epitopes capable of distinguishing normal Tg from carcinoma-derived Tg or Tg from benign thyroid diseases have been made for decades. Several studies investigated differences in Tg solubility, electrophoretic behavior, and iodine and carbohydrate content (38,41-43). These potential physicochemical changes have also been associated with differences in the antigenicity of tumor-derived Tg as compared with normal Tg. In 1984, Kohno and cols. showed that a certain monoclonal anti-Tg antibody profile was unable to distinguish between normal Tg and Tg from patients with Graves’ disease, follicular adenoma, or follicular carcinoma, but exhibited very weak binding to Tg from patients with papillary carcinoma (44). This distinction did not appear to be attributable to differences in Tg iodine content between each disease, but rather to potential minor alterations in the amino acid sequence and/or polysaccharide structure of the protein. In a more recent study, Magro and cols. showed that papillary carcinoma-derived Tg contained high levels of keratan sulfate, and suggested that this phenomenon could even be used to distinguish papillary cancer from benign thyroid diseases, in which expression of this heteropolysaccharide is negligible (45). In addition, Emoto and cols. found that papillary carcinoma-derived Tg contained chondroitin sulfate, a glycosaminoglycan found exclusively in human Tg, and exhibited very little sulfation as compared with Tg derived from normal tissue (33). These changes in glycosylation pattern are consistent with previous studies of other tumors and reinforce the hypothesis of involvement of these modifications both in carcinogenesis and in increased antigenicity of aberrant forms of Tg (46,47).

## TG HETEROGENEITY: CLINICAL APPLICATIONS AND FUTURE PERSPECTIVES

As noted at the start of this review, measurement of Tg levels in serum plays an essential role in the follow-up of patients with DTC after thyroidectomy. In this setting, increased Tg levels are indicative of residual or recurrent disease, whereas undetectable Tg levels and elevated TSH levels strongly suggest that the patient is disease-free.

However, serum Tg measurement is subject to interference due to the presence of heterophile antibodies (such as human anti-mouse antibody) or endogenous TgAb. The latter are much more prevalent in patients with DTC (25-30%) than in the healthy population (10%) (4). As mentioned before, the reason for this discrepancy is unknown; however, considering the wide range of posttranslational modifications present in tumor-derived Tg, one may speculate that these changes may contribute to increased antigenicity. Recent findings of Lupoli and cols. seem to support this hypothesis (48). In their study, TgAb from patients with DTC had a distinct specificity for different epitopes of Tg when compared with TgAb from patients with Hashimoto’s thyroiditis, possibly reflecting the presence of an abnormal Tg in patients with DTC (48). However, even the most modern assays available for Tg measurement cannot avoid this interference; i.e., both immunometric methods (immunoradiometric and immunochemiluminometric assays) and Tg recovery tests generally yield falsely low results when TgAb are present in the sample (49).

The presence of these endogenous anti-Tg antibodies in the follow up of DTC is an additional clinical challenge and may cause some degree of uncertainty either to the physician, regarding the status of the disease, or to the patient, causing anxiety, besides the increase in treatment costs due to the higher number of laboratory and imaging tests. Previous studies have shown that higher titers of anti-Tg antibodies are associated with higher risk of recurrence and have found that the higher the levels of these antibodies, the higher the chances of tumor recurrence (50-52). Although the presence of antibodies hinders the use of Tg as a follow-up marker, in some cases the serial TgAb measurements function as a surrogate marker (53). Thus, the majority of patients with DTC and positive TgAb tend to exhibit a progressive decline in titers of these antibodies, and a new increase in antibody levels may be considered a sign of tumor recurrence (52,53). However, anti-Tg antibody positivity itself does not correlate directly with worse prognosis, unless titers increase progressively. In fact, it is the trend of serum concentration of TgAb, i.e., if it is downward or upward, which will define patient outcomes (53). Regardless of the implications of this phenomenon, the reappearance of anti-Tg antibodies in these patients is an additional indicator of the increased antigenicity of tumor-derived Tg.

Another issue encountered in clinical practice is the wide variability in results when different methods are used for Tg measurement (4). As these methods may employ monoclonal antibodies that target distinct epitopes of Tg, it is not unusual to find marked disparities between Tg values during follow-up when different assays are used for Tg measurement (4,9). Furthermore, monoclonal antibodies are produced from normal thyroid-derived Tg, and strong evidence suggests that plasma-derived Tg, particularly that produced by tumors, may contain epitopes distinct from those present in tissue-derived Tg. This creates an additional problem, which is the possibility of false-negative Tg measurements in the presence of active disease (49,54).

Several methods have been proposed in an attempt to bypass this issue, such as measurement of Tg mRNA in peripheral blood (55), use of liquid chromatography tandem mass spectrometry (LC-MS/MS) to detect Tg in serum (10), detection of methylation markers (56), and serum measurement of TSH receptor mRNA (57), but none is sufficiently sensitive for clinical use. Additional strategies already used in the diagnostic investigation of other neoplasms, such as circulating tumor cell detection (58) and cancer cell exosome analysis (59), are very appealing, but have yet to become established methods in DTC.

One of the most promising attempts to escape the interference of TgAb on the Tg dosage is LC-MS/MS. In this technique, the entire serum protein is degraded with trypsin and the levels of Tg-specific peptides are used as a reference to quantify Tg (10). Although potentially specific, this analysis still poses difficult challenges to solve. Initially, this method has low sensitivity (detects Tg in the range of 0.5-1.0 ng/mL) compared with current immunoassays. Another challenge has been to enrich the sample under analysis with the trypsinized fragments of serum Tg from patients with DTC before analysis by LC-MS/MS, since tumor Tg does not necessarily would generate, after trypsin digestion, peptide sequences with the same charge/mass ratio of that from standard Tg used as reference.

Thus, the question remains whether polymorphisms in the Tg gene modifying the primary polypeptide sequence of the protein may affect the generation of target peptides to be identified by LC-MS/MS. In practice, this could really be a problem in view of recent studies that found two phenomena that may change the primary polypeptide sequence of Tg: different single-nucleotide polymorphisms in the Tg gene leading to an amino acid exchange (60) and somatic mutations in tumor cells (2.7%) in a significant number of patients with DTC (61). Tg gene may carry several polymorphisms and some of them have been associated with autoimmune thyroid disease (62). Besides, different TG RNA splicing forms from different human thyroid tissues have also been described in tumor samples and this kind of variation seems to be of high frequency, even in normal thyroid tissue (63).

In addition, any posttranslational modifications may also cause loss of peptides of interest, preventing their identification by LC-MS/MS and resulting in falsely low or even negative values in the presence of disease. It is important to notice that in order for the generation of appropriate peptides to be impaired, it is sufficient that any posttranslational modification produces a trypitc fragment with a different charge/mass ratio, thus preventing its recognition by LC-MS/MS analysis (10). An example for these interferences would be seen in one of two peptides specifically targeted for LC-MS/MS, VIFDANAPVAVR, which is considered well conserved and protected from posttranslational modification (64). This peptide (located at amino acid position 1579-1590 of thyroglobulin) contains an Asparagine in its structure, an amino acid that is glycosylated in normal human Tg (16). Although polymorphism at this position is rare (< 0.01%, rs141316336), other variations in the region flanking this peptide, and shortly beyond, could change the type of amino acid and then potentially interfere on the site for trypsin action.

Besides, even if a hypothetic patient with metastatic thyroid tumor produces a Tg with this exact peptide sequence but with no glycosylation at Arginine position, this peptide will not be detected by LC-MS/MS. In fact, a recent study by Spencer and cols. showed the limitations of Tg identification by LC-MS/MS; this strategy, for example, fails to detect serum Tg levels in nearly one fourth of patients with positive TgAb and persistent/recurrent disease (65). In that report, the authors proposed two theories to explain this fail: the presence of polymorphisms in tumor-derived Tgs preventing the generation of target peptide; or an increased clearance of Tg-anti-Tg complexes that has been hypothesized for a long time (66-68). However, it is important to note that despite initial difficulties, LC-MS/MS already represents a major advance over immunoassays in which the disease is not detected in more than 98% of cases (65). One possibility to overcome this new challenge may be the development of a wider panel of proteotypic peptides, which could increase the sensitivity and specificity of LC-MS/MS assays, thus reducing the likelihood of false-negative results.

Despite the aforementioned issues, Tg measurement remains the cornerstone of follow-up of patients with DTC. Therefore, new techniques that can enhance its detection are essential. Modern methods, particularly those involving proteomics and mass spectrometry analysis (69), may hold the key to the identification of anomalous tumor-derived forms of Tg and thus enable the development of more specific assays that can contribute to diagnosis, follow-up, and prognostication of DTC.
